# A fault-tolerant aware scheduling method for fog-cloud environments

**DOI:** 10.1371/journal.pone.0223902

**Published:** 2019-10-17

**Authors:** Abdulaziz Alarifi, Fathi Abdelsamie, Mohammed Amoon

**Affiliations:** 1 Department of Computer Science, Community College, King Saud University, Riyadh, Saudi Arabia; 2 Department of Electronics and Electrical Communications, Faculty of Electronic Engineering, Menoufia University, Menouf, Egypt; 3 Department of Computer Science and Engineering, Faculty of Electronic Engineering, Menoufia University, Menouf, Egypt; University of Catania, ITALY

## Abstract

Fog computing is a promising technology that leverages the resources to provide services for requests of IoT (Internet of Things) devices at the cloud edge. The high dynamic and heterogeneous nature of devices at the cloud edge causes failures to be a popular event and therefore fault tolerance became indispensable. Most early scheduling and fault-tolerant methods did not highly consider time-sensitive requests. This increases the possibility of latencies for serving these requests which causes unfavorable impacts. This paper proposes a fault-tolerant scheduling method (FTSM) for allocating services’ requests to the most sufficient devices in fog-cloud IoT-based environments. The main purpose of the proposed method is to reduce the latency and overheads of services and to increase the reliability and capacity of the cloud. The method depends on categorizing devices that can issue requests into three classes according to the type of service required. These classes are time-sensitive, time-tolerant and core. Each time-sensitive request is directly mapped to one or more edge devices using a pre-prepared executive list of devices. Each time-tolerant request may be assigned to one or more devices at the cloud edge or the cloud core. Core requests are assigned to resources at the cloud core. In order to achieve fault tolerance, the proposed method selects the most suitable fault-tolerant technique from replication, checkpointing and resubmission techniques for each request while most existing methods consider only one technique. The effectiveness of the proposed method is assessed using average service time, throughput, operation costs, success rate and capacity percentage as performance indicators.

## 1 Introduction

Cloud computing systems introduce inexpensive services to customers primarily in terms of storage and computing. However, the centralized architecture of the cloud can exhaust the available bandwidth and increase the service time. In some instances, the large number of customers’ requests and the large distance between data center core and customers can greatly increase network traffic and then some services could be delayed or cut off. Additionally, this large distance gives rise to high service latency, which is not advisable for time-sensitive and critical safety applications such as healthcare, manufacturing and smart cities. In addition, applications that depend on the Internet of Things (IoT), which represents the next generation of networks, require real-time responses. Some IoT applications have enormous amounts of generated data collected from sensors attached to things and these data could exhaust the bandwidth of the network. Thus, classical cloud systems are not sufficient to meet the requirements of these applications because of the broad distance between IoT devices and the processing and storage elements at the cloud core. Consequently, there is a need for a computing model that distributes cloud services away from the cloud core to the cloud edge in order to avoid delays caused by moving data to the cloud core [1].

Fog computing, known as computing at the cloud edge, appears as a computing model to face and resolve the above issues. The model of fog computing aims to minimize the burden of the data centers by providing service support to the geographically separated and real-time services. In addition, the model acts as an interface that manages the virtual environment between the cloud core and the other resources located at the edge of the cloud. In summary, it extends the cloud computing model by serving some customers’ requests by devices located at the cloud edge instead of sending them to be executed at the cloud core [2].

The incident of failures is one of the most important challenges that restrict the deployment of fog computing. In IoT-based environments, as in fog-cloud computing, there is a great number of devices connected to the cloud at its edge. It is estimated that by 2020, 40% of cloud data will come from these devices [3]. These devices can request services and some of them have the ability to serve some requested services. In such environments, the occurrence of failures is popular because of the great dynamic and heterogeneity nature of the IoT devices and the interactions between them and the external environment [4]. The effect of these failures ranges from minor influences resulting from delaying non-critical tasks which represent 70% of the cloud workload to major damages resulting from delaying critical tasks which represent about 30% of the cloud workload [5]. Both the cloud provider and the cloud customer are suffered due to failures incidents. Customers cannot get their services at the desired time and providers can lose their reputation and money.

The LLOYD’s Emergency Risk Report of 2018 introduced a deep analysis of the impact of service disruptions on various sectors of the economy such as insurance, information, manufacturing, etc. The report reveals that infrastructure failures, including hardware and software components, are among the main reasons for services’ outages. In addition, the outage period from 3 to 6 days would bring out a loss from $6.9bn to $14.7bn. Consequently, cloud vendors should prepare policies to prevent failures occurrence to the most possible extent and to transparently restore in case of failures events. Table 1 shows some details of the outages occurred in 2018 for common cloud vendors [6].

**Table 1:** Some outages of 2018.

Vendor	Outage	Description	Date	Period
**YouTube**	Inaccessible	A blank page appeared for users without videos and network problem message appeared for mobile applications	Oct. 17, 2018	30 Minutes
**Microsoft Azure**	Network and Storage availability problems in Europe	Some network and storage resources cannot be accessed by customers because of the failure of a heat-down control system	June 17–18, 2018	5 Hours
**Amazon Web Services**	A data center was down at North Virginia	Some users were down and not all their data could be restored because of power failures of some servers and networking devices.	May 31, 2018	30 Minutes
**Microsoft Office 365**	E-mail accounts were locked in Asia, Europe and the USA	Some customers cannot access their e-mails and cannot log in to Skype because of some security concerns	April 6, 2018	On day
**Google**	A database malfunction	Some customers could not connect with their data or face a high latency to connect with it.	Feb. 15, 2018	On day

In result, the failures cause latency for services, especially time-sensitive, which could prevent obtaining the interest of the fog computing deployment. The main contributions of this work are:

- Proposing an architecture for providing services in fog-cloud environments. The architecture has three layers: the cloud core layer, the edge layer and the things layer. Models for fog-cloud, time and failure are also introduced within the proposed architecture.
- Proposing a scheduling method for allocating services’ requests of devices located at the cloud edge to the most appropriate actuators. The method classifies the requester devices according to the response time-sensitivity of their requests.
- Providing the fault tolerance service for the fog-cloud devices through the employment of the following three fault-tolerant techniques: replication, checkpointing and resubmission. The most suitable technique is selected for each request based on the request type and the failure information available about the allocated device.
- Proposing a checkpointing algorithm with a variant checkpoint interval. The concept of the algorithm is based on the standard deviation of the operation time between failures. In addition, the algorithm stores the status of the device on the backup device that will complete the execution of the request when the primary device fails.
- Providing replication with an adaptive number of replicas according to the request’s category and the failure probability of the assigned device to serve the request.

The next sections of the paper are as follows: Sect. 2 presents the previous work. Sect. 3 presents the models used. In Sect. 4, the components of the proposed architecture are presented with their functions and the proposed method is explained in details. Sect. 5 shows and discusses simulation results. Conclusions and future research suggestions are presented in Sect. 6.

## 2 Related work

Fault tolerance had become a mandatory aspect for fog-cloud computing environments and a considerable number of methods have been proposed by a great number of researchers in order to realize it. These methods are distinguished as proactive or reactive. Proactive methods include some preventive actions. They are applied during the scheduling and before starting the service of the request through the anticipation of the devices that may fail and avoidance assigning services’ requests to them. This means that the reaction against faults is implemented before starting the request’s service in order to prevent or avoid the failure incident. In these methods, the status of the cloud is continuously observed in order to collect the required information to make preventive decisions. Rejuvenation and self-healing are among the most common proactive methods deployed in cloud computing.

In contrast, reactive fault-tolerant methods take their reaction against a failure after it has occurred. Here, reactions are implemented after starting the request’s service. Hereby, the status of the cloud is continuously observed in order to detect failures. Reactions could be done through replication, checkpoint, and resubmission [7]. Resubmission could cause a violation of the service level agreement due to the delays experienced by the requests in order to be fulfilled. Checkpointing could cause delays and exhaust cloud storage resources. Although replication is the most exhaustive reactive strategy for resources, it is the most used one [8]. This is because the replication method reduces lateness nearly to zero. Amazon Ec2 applies replication through the auto scaling group that allows creating and running multiple copies of the same application, simultaneously [9]. Also, Microsoft Azure uses replication for tolerating faults [10].

Despite there is a large number of fault-tolerant methods proposed for grid [11] and cloud computing [12], fault tolerance is still a great challenge in fog-cloud computing environments and there is a little research work that considers it [13]. Goiri et al. [14] have presented a checkpoint-based fault tolerant method that minimizes the storage time needed to preserve checkpoints. Their method saves only the values of parameters changed. J. Cao et al. [15] have proposed a checkpoint-based method for long jobs and their method depends on assigning priorities for jobs. In [16], S. Abdulhamid and M. Latiff have developed a scheduling algorithm that considers checkpointing and migration to deal with failures. T. Louatia et al. [17] have developed a checkpoint method based on hash tables and storing checkpoints information in a distributed manner.

P. Das and P. Khilar [18] have developed a replication scheme that depends on the variation of programs on different virtual machines. The scheme can enhance reliability by avoiding the allocation of tasks to resources with a high failure rate. Alhosban et al. [19] have proposed a method that can select between resubmission and replication to achieve fault tolerance. User needs and service criticality are the main criteria for selection. Also, S. Saranya et al [20] have developed a fault-tolerant scheme that selects between resubmission and replication according to task priority. X. Zhu et al [21] have addressed the problem of employment the primary backup scheduling for real-time workflows in cloud computing. Their mail goal was to tolerate faults and improve utilization. For this, they have developed a dynamic scheduling algorithm to tolerate faults for real-time workflows.

V. Souza et al [22] have presented a linear programming model to evaluate the deployment of reactive and proactive recovery methods in the fog computing environment. Their goal is to absorb single commodity failures. K. Wang et al [23] have developed a mechanism to enhance the reliability of data transfer in healthcare fog computing. Their mechanism depends on Directed Diffusion technique and Limited Flooding method. Additionally, they developed an adaptation scheme for controlling parameters of the developed mechanism according to the current status of the fog. In [24], K. Dantu et al have presented reliable software architecture for Android-based systems in fog environments. Their model can be easily adapted to other mobile operating systems.

In [4], authors have proposed a protocol of four steps to manage failures. Their protocol saves the state of the services using checkpoints combined with message logging. Then, it observes the environment to find out and reports failures. If a failure is expected to occur the protocol can take an appropriate decision to prevent it. In case of failure, the protocol notifies the dependent entities to perform reconfiguration actions. Finally, the protocol determines the suitable actions for recovery.

R. Oma et al [25] have presented a fault-tolerant approach for tree-based fog architectures. Their approach comprises the employment of replication and non-replication techniques. In replication, multiple fog nodes serve the same requests. In non-replication, a different fog node replaces the faulty one.

It is obvious from the above review that most fault-tolerant techniques were developed for the cloud computing environment and a little number of researchers considers fog-cloud computing. Most of the previously developed techniques did not consider the type of requesting devices from the perspective of the time sensitivity. However, the IoT time-sensitive devices need a highly reliable and efficient computing environment. In addition, most methods consider only one technique to achieve fault tolerance, e. g. replication or checkpointing or consider one of them besides resubmission. Also, they assume a constant number of replicas in case of replication and constant checkpoint interval in case of checkpointing. This could exhaust the resources and reduce the performance of the computing environment. Thence, scheduling and fault tolerance are still considerable challenges in fog-cloud computing environments.

## 3 System models

### 3.1 Fog-cloud model

The fog-cloud computing environment comprises a set of cloud data centers located at its core and a set of fog stations located at its edge. Table 2 shows a summary of all nomenclatures used in this paper.

**Table 2:** Nomenclatures summary.

nomenclature	Description
*c*	Data center
*V*_*c*_	Set of virtual machines
*v*_*c*_	Virtual machine
$R_{v_{c}}$	Set of resources owned by virtual machine v_c_
*F*	Set of fog stations
*f*	Fog station
*n*	Number of virtual machines
*m*	Number of fog stations
*I*_*f*_	Set of IoT devices owned by fog station *f*
*d*_*f*_	Device owned by fog station *f*
$I_{f}^{ser}$	Set of actuator devices owned by fog station *f*
$I_{f}^{sen}$	Set of devices owned by *f* and can an issue time-sensitive requests
*q*	Request
*τ*_*q*_	The anticipated service time of *q*
*τ*_*ql*_	The time spent in moving the request *q* and its serving results through the cloud
*τ*_*qs*_	The time spent in serving the request *q* by the cloud resources
*τ*_*qw*_	The waiting time encountered by the request *q* in the cloud
*Z*_*q*_	The size of the request *q*
*Z*_*r*_	The size data resulting from serving the request *q*
*B*	The average of the available network bandwidth
*I*_*q*_	The number of instructions of the request *q*
*S*	The average processing rate of the resource
*τ*_*qsh*_	The time the request *q* will wait until it is assigned to the resource
*τ*_*a*_	The time spent from receiving the request *q* until the availability of data
*τ*_*b*_	The time the request *q* will wait until the allocated resource finishes its current work
*μ*_*d*_(*t*)	The mean failure rate of device *d*
*n*(*T*)	The anticipated number of failures during a certain time period from 0 to *T*
*P*(*p*|*μ*_*d*_)	The probability of the occurrence of *p* failures for device *d*
*p*	Number of failures
$L_{d_{i}}$	Actuator list that can serve the requests of the device *d*_*i*_
*k*	The number of backup devices
*OTBF*_*d*_	The operation time between failures of device *d*
$t_{d}^{lf}$	The operation time from the last failure of a device *d*.
$t^{q_{j}}$	The service time required by the request *q*_*j*_
$c^{q_{j}}$	The cost rate required by the request *q*_*j*_
$p^{q_{j}}$	The amount of power limit for the request *q*_*j*_
$t_{x}^{q_{j}}$	The service time when serving *q*_*j*_ on the device *x*
$c_{x}^{q_{j}}$	The cost rate when serving *q*_*j*_ on the device *x*
$p_{x}^{q}$	The amount of power consumed when serving *q*_*j*_ on the device *x*

Each data center *c* owns a set *V*_*c*_ of virtual machines, which can be represented as:

$$V_{c}=\{v_{c1},v_{c2},\ldots ,v_{cn}\}.$$ (1)

Each virtual machine *v*_*c*_ owns a set $R_{v_{c}}$ of *k* resources that may belong to one or more servers. These resources may include CPUs, memory storage, network resources, etc. It can be represented as:

$$R_{v_{c}}=\{r_{v_{c}}^{1},r_{v_{c}}^{2},\ldots ,r_{v_{c}}^{k}\}.$$ (2)

Each cloud has a set *F* of *m* fog stations located at its edge, which is represented as:

$$F=\{f_{1},f_{2},\ldots ,f_{m}\}.$$ (3)

Each fog station *f* owns a set *I*_*f*_ to *h* IoT devices connected to it. Each device may have a set of sensors and/or actuators. Each device can send a service’s request to be served either at the cloud core or at the cloud edge. The *I*_*f*_ can be represented as:

$$I_{f}=\{d_{f}^{1},d_{f}^{2},\ldots ,d_{f}^{h}\}.$$ (4)

Some devices $I_{f}^{ser}$ of the *I*_*f*_ devices play the role of actuators and then they can serve some requested services. The $I_{f}^{ser}$ can be represented as:

$${I_{f}^{ser}=\{d_{f}^{i}\},\;\text{where}\;I}_{f}^{ser}\subseteq I_{f}\;and\;1\leq i\leq h.$$ (5)

In addition, some devices $I_{f}^{sen}$ can issue time-sensitive requests. The $I_{f}^{sen}$ can be represented as:

$${I_{f}^{sen}=\{d_{f}^{j}\},\;\text{where}\;I}_{f}^{sen}\subseteq I_{f}\;and\;1\leq j\leq h.$$ (6)

### 3.2 Time model

For each request *q*, the anticipated service time is *τ*_*q*_ and it is determined by

$$\tau_{q}=\tau_{ql}+\tau_{qs}+\tau_{qw},$$ (7)

where *τ*_*ql*_ is the total amount of time spent in moving the request *q* and its serving results through the cloud, *τ*_*qs*_ is the amount of time spent in serving the request *q* by the cloud resources and *τ*_*qw*_ is the total amount of waiting time encountered by the request *q* in the cloud. The amount of *τ*_*ql*_ can be determined as follows:

$$\tau_{ql}=\frac{Z_{q}+Z_{r}}{B},$$ (8)

where *Z*_*q*_ is the size of the request and its required data, *Z*_*r*_ is the size of the data resulting from serving the request and *B* is the average of the available network bandwidth. The value of *τ*_*s*_ can be determined as follows:

$$\tau_{qs}=\frac{I_{q}}{S},$$ (9)

where *I*_*q*_ is the number of instructions of the request *q* and *S* is the average processing rate of the resource that will serve the request. It is usually measured in MIPs. The amount of *τ*_*w*_ is defined as the time elapsed from sending the request *q* until the assigned device or machine starts to serve it. It can be determined as follows:

$$\tau_{qw}=\tau_{qsh}+Max(\tau_{qa},\;\tau_{qb}),$$ (10)

where *τ*_*qsh*_ is the time the request *q* will wait until it is assigned to the resource that will serve it, *τ*_*a*_ is the time spent from receiving the request *q* until the availability of data required to start serving it and *τ*_*b*_ is the time the request *q* will wait until the allocated resource finishes its current work.

### 3.3 Fault model

In the fog-cloud environment, failures became a popular event. Failures appear in consequence of software or hardware malfunctions. As a result of failures, some cloud core hosting servers may partially or completely stop working and therefore all or some of their hosted virtual machines stop serving the requests.

At the edge of the cloud, some connected devices may fail and consequently, the requests allocated to these devices undergo latencies. Generally, failures stochastically happen in fog-cloud environments. Failures modeling depends on the time interval spent between two successive failures which can be represented as a random variable of the Poisson probability distribution as in [26], [27]. This reveals that the number of failures of devices will not be the same for any two independent time periods.

Assuming that the mean failure rate is determined, then the number of failures occurred for a device during a certain time period would follow the non-homogeneous Poisson probability distribution. Thus, the anticipated number of failures during a certain time period from 0 to *T* is given by:

$$n(T)=\int_{0}^{T}\mu_{d}(t)dt,$$ (11)

where *μ*_*d*_(*t*) is the mean failure rate of a device *d*. The probability of the occurrence of *p* failures for device *d* during the time period from 0 to *T* given *μ*_*d*_ is:

$$P(p|\mu_{d})=\frac{\mu_{d}^{p}}{p!}e^{-\mu_{d}},\;0\leq P(p)\leq 1.$$ (12)

## 4 The proposed method

Applying fault tolerance in the fog-cloud computing environment is more complex than applying it in the traditional cloud computing environment. This is because, in addition to the high dynamism and heterogeneity, the fog-cloud computing environment has two levels of computing: computing at the core of the cloud and computing at the edge of the cloud. These two levels of computing should be considered when making decisions related to scheduling and fault-handling. In addition, fog or edge devices are used mainly for serving time-sensitive requests of significant sectors such as healthcare, smart cities, manufacturing, etc. Furthermore, rapid reactions could improve the performance and security levels in many fields of the public sector. Thus, these issues should be carefully considered when developing scheduling and fault-handling strategies for fog-cloud computing environments.

### 4.1 The employed architecture

**Fig 1:**
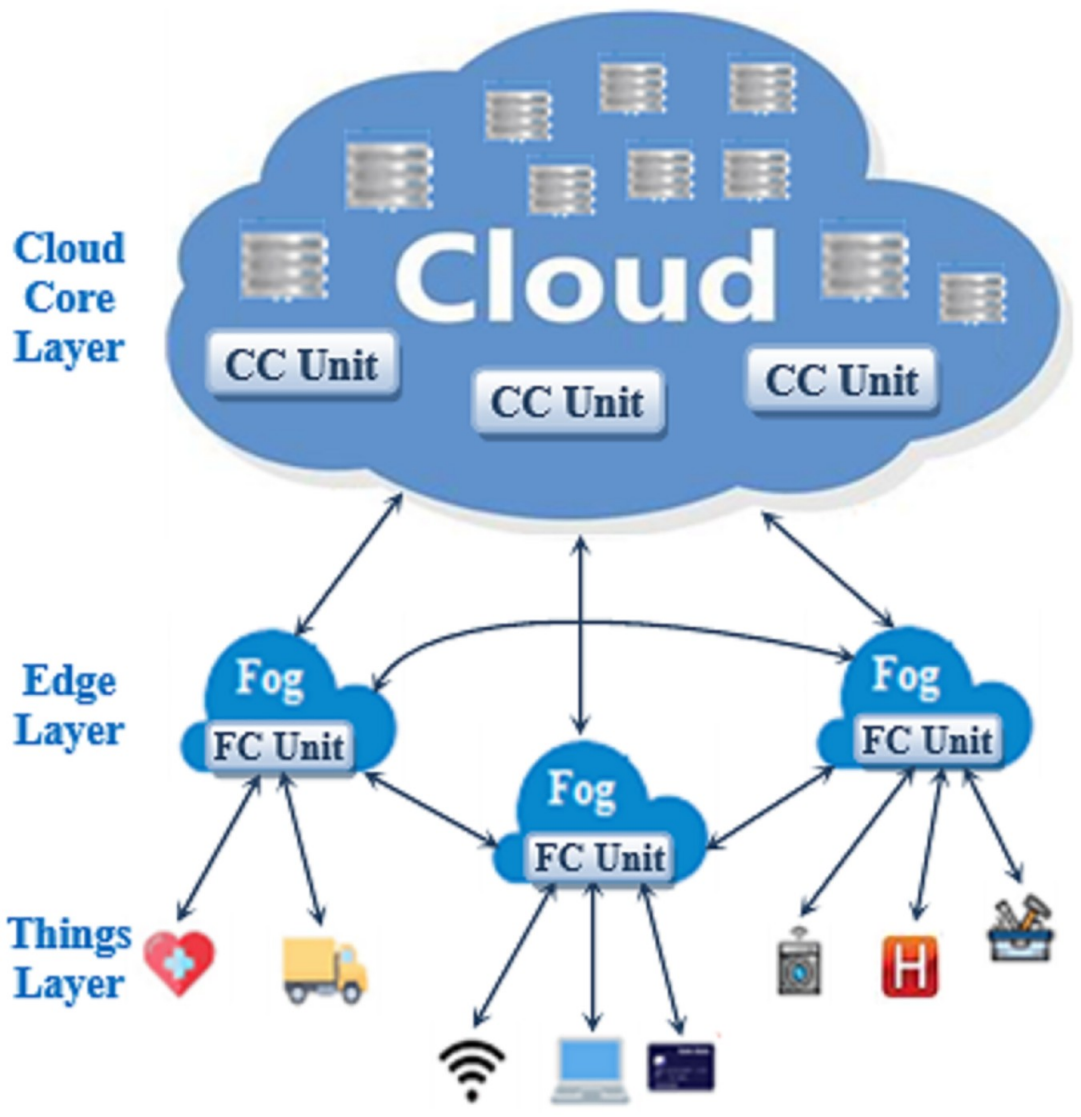
The fog-cloud environment.

The architecture and the intercommunication among components of the fog-cloud computing environment used in this work are displayed in Fig 1. The architecture has three layers: the cloud core layer, the edge layer and the things (devices) layer. The cloud core layer includes cloud data centers with different arrangements of multiple virtual machines in each data center. Additionally, it includes the servers used for constructing and running the virtual machines. Besides servers and virtual machines, it has a set of Cloud Control Unit (CC Unit) which can receive requests that cannot be served by the devices located at the edge of the cloud. Each CC Unit can allocate each incoming request to the most suitable virtual machine located at its data centers. The decision of allocation depends on the required level of the service in order to satisfy both the requester and the provider of the service. The CC Unit monitors each request until completion and returns feedback to the requester.

**Fig 2:**
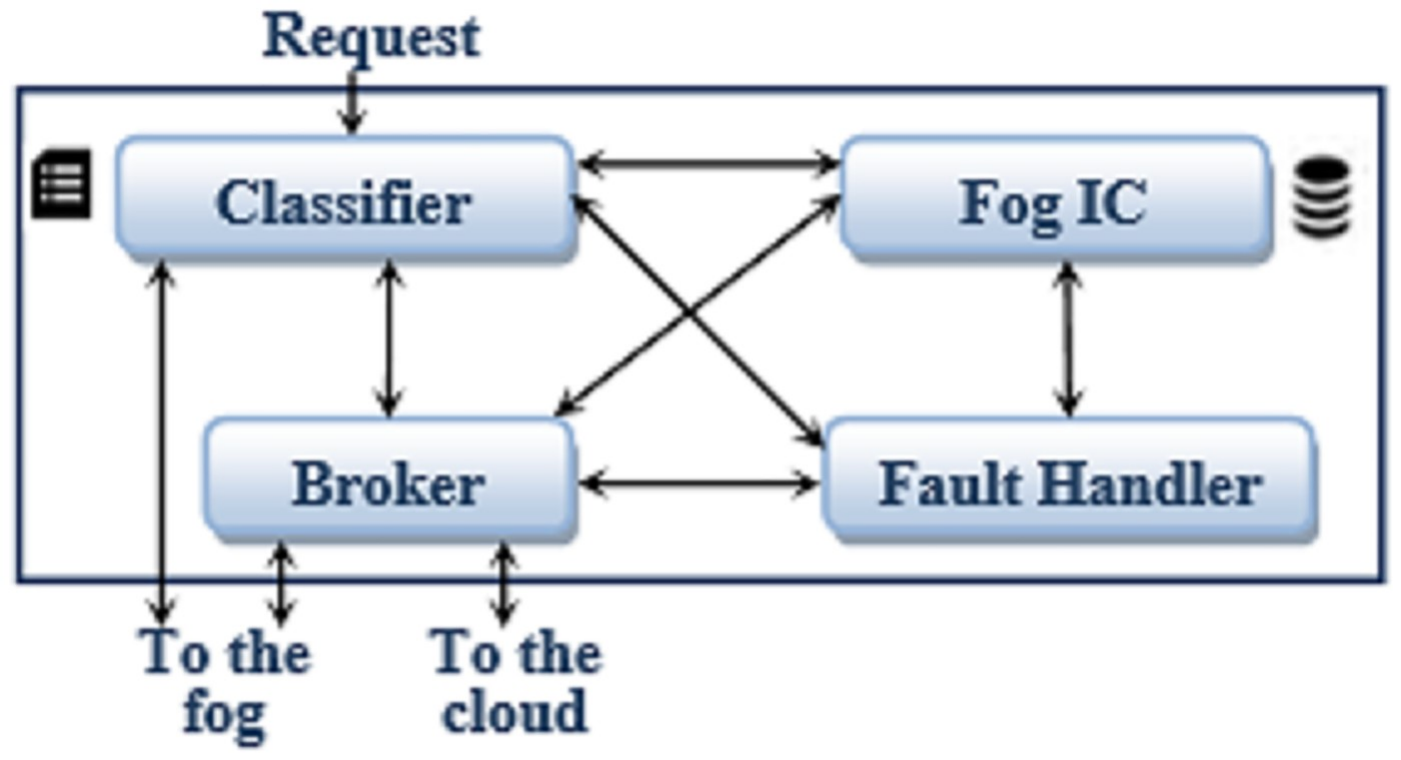
The FC internal construction.

The edge layer comprises fog stations, simply called fogs. Each fog has a Fog Control Unit (FC Unit) that can receive the requests with their service requirements from the IoT devices connected to the fog. Then, the FC Unit decides whether each request will be served by the devices located at the things layer or by the virtual machines located at the cloud core. This decision is firstly based on the device that issues the request, the required quality level of the service and the availability of the resources required to fulfill the request. In case the things layer is selected to serve the request, the FC Unit will have the responsibility of determining the device that will serve the request. The allocated device may be located at the same fog or at another fog. The internal structure of the FC Unit and intercommunications between its modules are displayed in Fig 2. The FC Unit has four modules: Classifier, Broker, Fog Information Center (FOG IC) and Fault Handler.

### 4.2 The FTSM

This section describes in details the logic of the proposed FTSM method. The logic of the method is distributed among the Classifier, Broker and Fault Handler modules of the employed architecture.

The Classifier monitors the devices attached to the fog and categories them into three classes according to the type of the service’s request each device can issue. The first class, called time-sensitive, includes devices that could issue time-sensitive requests. These requests need a very distinctive and quick service since delaying the service of these requests could cause harmful damages. In most cases, these requests should be served at the same fog of the requester’s device or at another nearby fog. The second class, called time-tolerant, includes devices that could issue time-tolerant requests which do not have special needs related to the service’s time. Therefore, it can be served by devices attached to the fogs or by virtual machines located at the cloud core. The third class, called core, includes devices that could issue requests that should be served by virtual machines located at the cloud core because there are no sufficient resources to serve them at the cloud edge. The main logic of the classifier depends on using the decision tree classification algorithm which is based on if-then rule.

For each requester device *d*_*i*_ in the first class (time-sensitive), the Classifier sets up an actuator list $L_{d_{i}}$ of *n*_*i*_ devices that can serve the requests of *d*_*i*_ within the desired time limits as follows:

$$L_{d_{i}}=\{d_{0},\;d_{1},\ldots ,d_{n_{i}-1}\}\;where\;L_{d_{i}}\subseteq I_{f}^{ser}.$$ (13)

**Fig 3:**
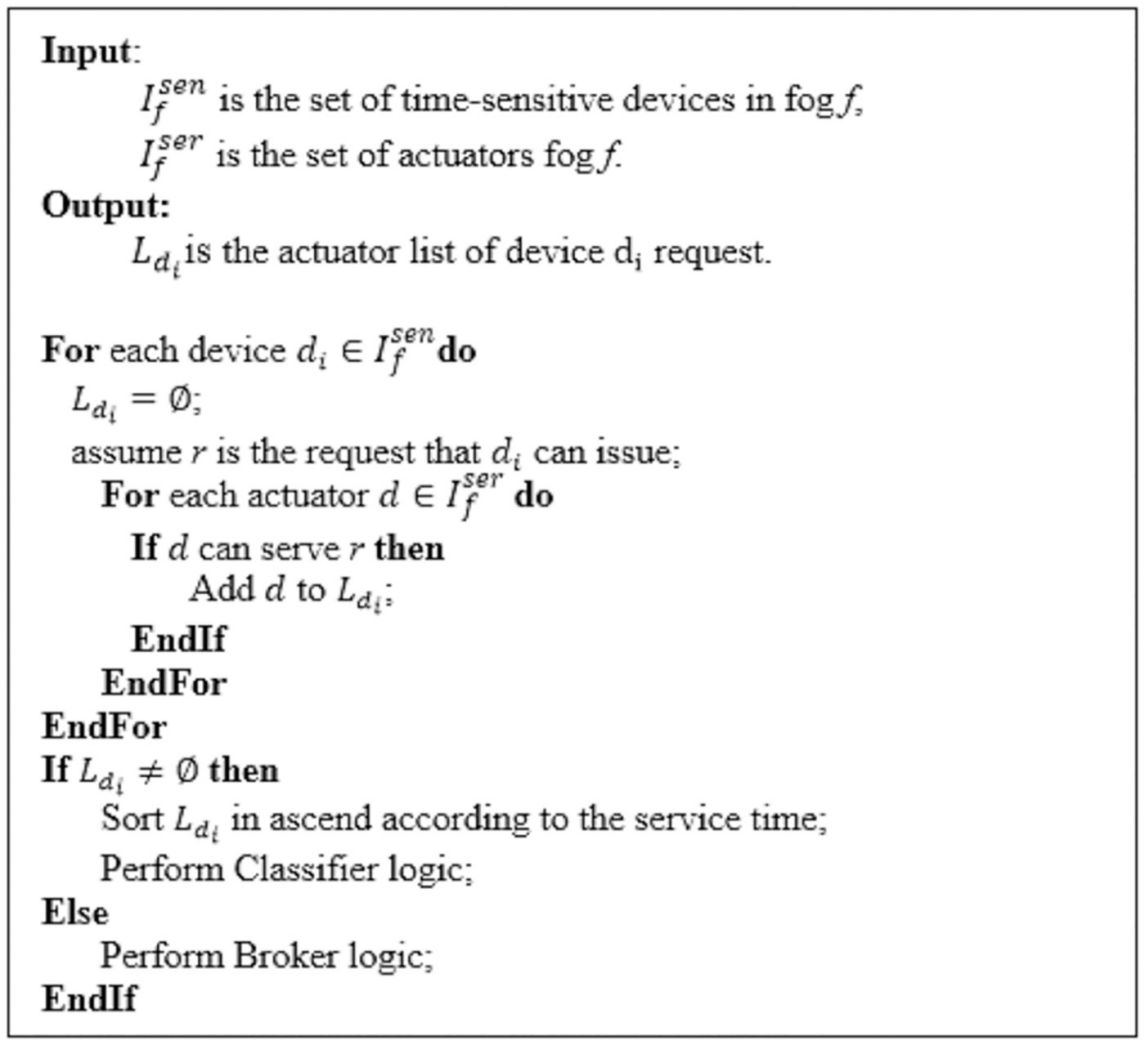
The algorithm for constructing the actuator list.

Fig 3 presents the algorithm steps of constructing the actuator list $L_{d_{i}}$ for each device in the time-sensitive class. The list is ordered in ascend according to the anticipated response time expressed by Eq (7). Also, it is periodically updated through consulting the Fog IC module.

**Fig 4:**
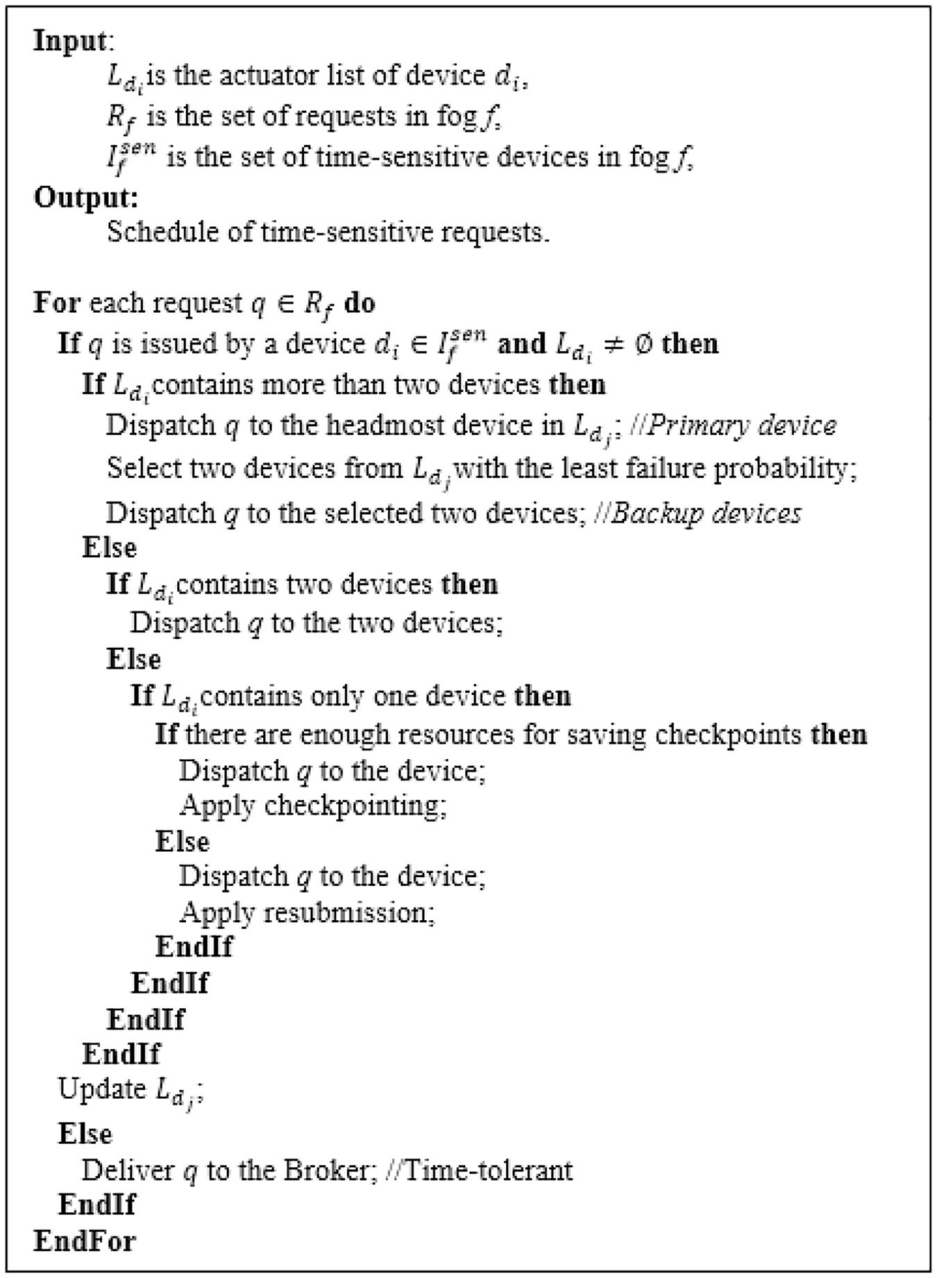
The Classifier main logic.

Now, when a device *d*_*i*_ associated with the fog *f* issues a request, the Classifier receives it. If *d*_*i*_ is a time-sensitive device the Classifier will directly dispatch it to the first *k* devices in the pre-prepared actuators list $L_{d_{i}}$. Then, the Classifier updates $L_{d_{i}}$. Thus, there are no scheduling overheads associated with each time-sensitive request because a pre-prepared list $L_{d_{i}}$ of sufficient devices for serving the request is already set up. This means that the time overheads expended in the request scheduling, denoted as *τ*_*sh*_ in Eq (10), will be avoided because scheduling was done before the advent of the request. Fig 4 indicates the steps of Classifier’s algorithm.

The main role of the Fault Handler module is to provide a fault-tolerant service for each request by selecting the relevant fault tolerance technique. The replication technique should be employed only if there is more than one device that can serve the request. In addition, it should not be employed for all types of requests because this will not be an economic desire. For time-sensitive requests, replication is applied through serving each request by *k* + 1 devices, simultaneously. These *k* + 1 devices comprise the primary allocated device besides a set of *k* backup devices. The first incoming response from any device of the *k* + 1 devices is considered to be sent to the request’s owner. The other devices will be informed to stop working on the request. Determining the value of *k* is a challenging task because extra copies could exhaust the cloud resources and could cause money lost from the provider perspective. On the other hand, the underestimated value of *k* may cause resubmission or rescheduling of requests, which is not suitable for time-sensitive requests. So, it is important to choose a reasonable value of *k* for each request.

Most replication-based methods determine only one additional backup device for each request and they select it according to its response time. However, the backup device may have a bad failure history. In this work, the backup device is selected so that it can fulfill the request within the time limits with the lowest probability of failure. Each time-sensitive request is considered to need a high level of reliability. Consequently, two replicas (*k* = 2) are assigned for each time-sensitive request. However, only one replica will be assigned for each time-tolerant request.

If there is only one device that can serve the request, checkpointing will be applied. In checkpointing, the state of request execution is saved to stable storage periodically. In the failure case, the service is resumed from the last checkpoint either on the same device or on another one. Here, the challenge is the selection of the checkpoint interval, which is the time between two consecutive checkpoints.

**Fig 5:**
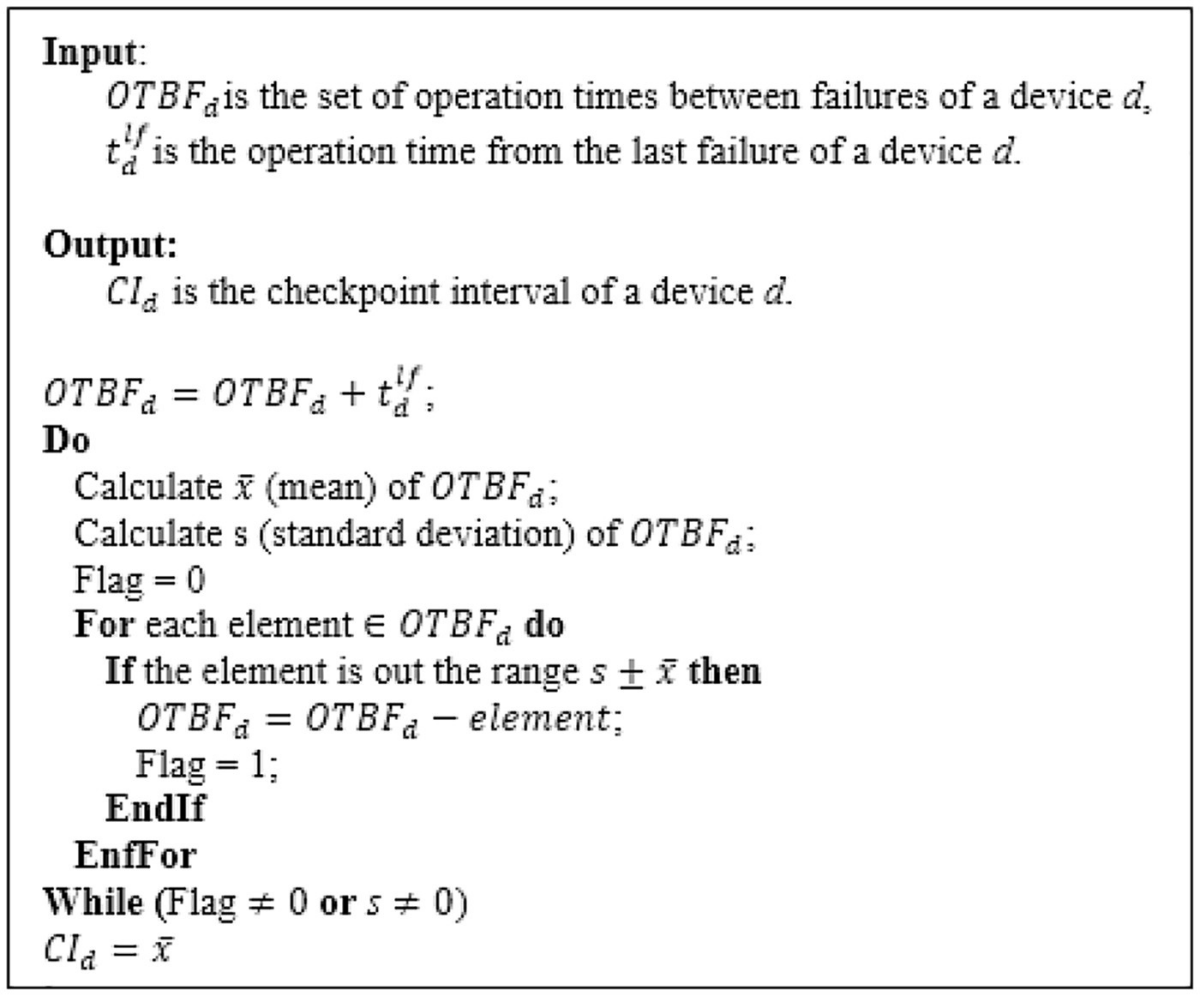
The checkpoint interval algorithm.

Most previous research works have assumed a constant checkpoint interval for each device. In this work, the checkpoint interval of a device *d* varies and it is determined based on the standard deviation of the operation time between failures (OTBF). Fig 5 shows the algorithm used to calculate the checkpoint interval for a device *d*. The calculated interval of the checkpoint tends to be close to the most repeated values of the time between failures. For each device, the value of this interval is updated either when serving an assigned request is completed or when a failure occurs. In addition, the status of the device is stored on the device that will complete the execution of the request in case the primary device fails for a long time. So, migration and time overheads could be minimized.

If the request is time-tolerant, the Classifier will deliver it to the Broker, which schedules it to a suitable resource. In order to accomplish its mission, the Broker consults the Fog IC module to get the required status information of edge devices in order to prepare the list $S_{q_{j}}$ of devices that can serve the request *q*_*j*_. Therefore, the Broker selects devices for serving the request from the $S_{q_{j}}$ list. The selection is based on the priority degree of each request. The priority degree is determined according to the requirements of the request such as the response time, the required quality of the service and the financial cost. Also, it is determined according to the conditions of the cloud such as power consumption and availability.

Here, the Fault Handler module also selects one of the following fault tolerance strategies: replication, checkpointing and resubmission. Replication is applied if there are multiple resources that can serve the request. If there are only one resource and enough resources for saving checkpoints, checkpointing will be applied. Otherwise, resubmission is applied.

**Fig 6:**
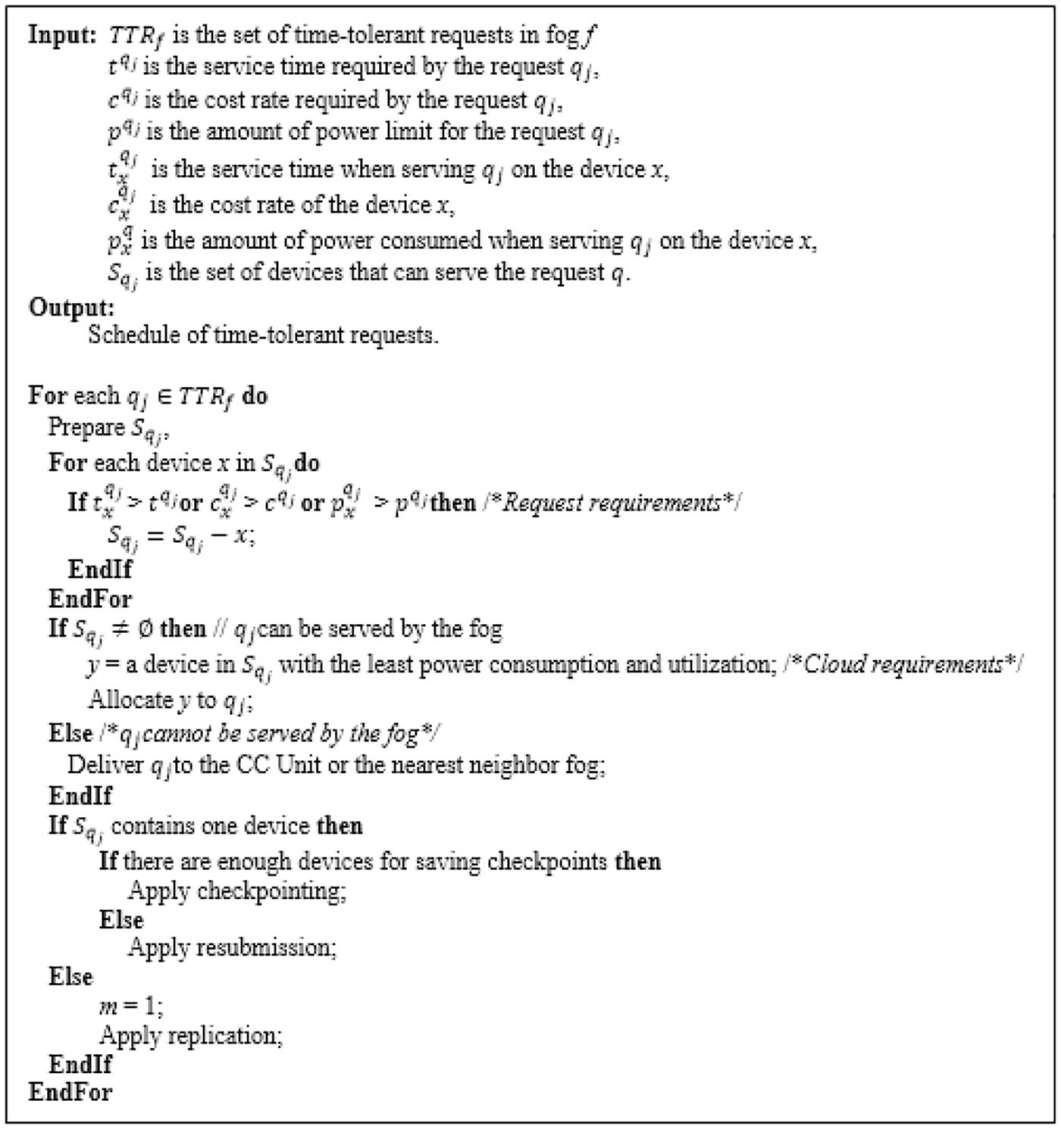
The Broker main logic.

Therefore, the Broker dispatches the request to the selected edge resource. If there are no resources that can serve the request at the current fog of the Broker, the Broker delivers it to the CC Unit or to the FC Unit of the nearest neighbor fog. The algorithm shown in Fig 6 describes the main logic of the Broker.

## 5 Performance evaluations

In this section, the performance of the proposed method is evaluated and the obtained simulation results are discussed. Firstly, the simulation environment and the setup of experiments are presented. Then, the results of the experiments are presented, discussed and analyzed.

### 5.1 Simulation setup

Most researchers use simulators in order to evaluate the performance of their proposed approaches and methods. The motivation behind that is the difficulty of finding real systems for their tries. There is a little set of existing simulators such as iFogSim [28], RECAP [29] and MyiFogSim [30] for modeling Fog environments. Although iFogSim simulator, developed by H. Gupta et al, is the most common one and it supports simulation of many aspects it does not support simulation of fault-tolerant techniques. Consequently, some software packages should be produced to reinforce the simulation of fault-tolerant methods.

In our experiments, we simulate a fog-cloud environment with 5 data centers and 20 fog stations. Each fog station has a number of directly connected heterogeneous devices ranges from 100 to 2000 devices. The size of data to be processed for each request ranges from 0.1 MI to 100 MI. The rate of requests arrival is assumed to be random in the range from 100 to 300 per minute. In order to simulate the failure environment, failures are injected in the computing environment with random numbers. This number of injected failures follows the Poisson probability distribution.

For evaluation purposes, the proposed FTSM method is compared against the method introduced in [4] which is a checkpoint-based scheduling method and for simplicity, we will call it CKM. In addition, the FTSM method is compared against the method introduced in [15] which is a replication-based method scheduling and for simplicity, we will call it RM. This method focused on the conditions of the fog environment like dynamism, heterogeneity and external interactions and it employed checkpoint as the fault tolerant technique. However, the proposed FTSM focuses on the type of service needed and it employed multiple fault-tolerant techniques.

### 5.2 Results

The selected performance indicators to evaluate the performance of the FTSM method should satisfy both cloud customers and providers. The indicators include average service time, throughput, operation costs, success rate and capacity percentage.

#### 5.2.1 Average service time

**Fig 7:**
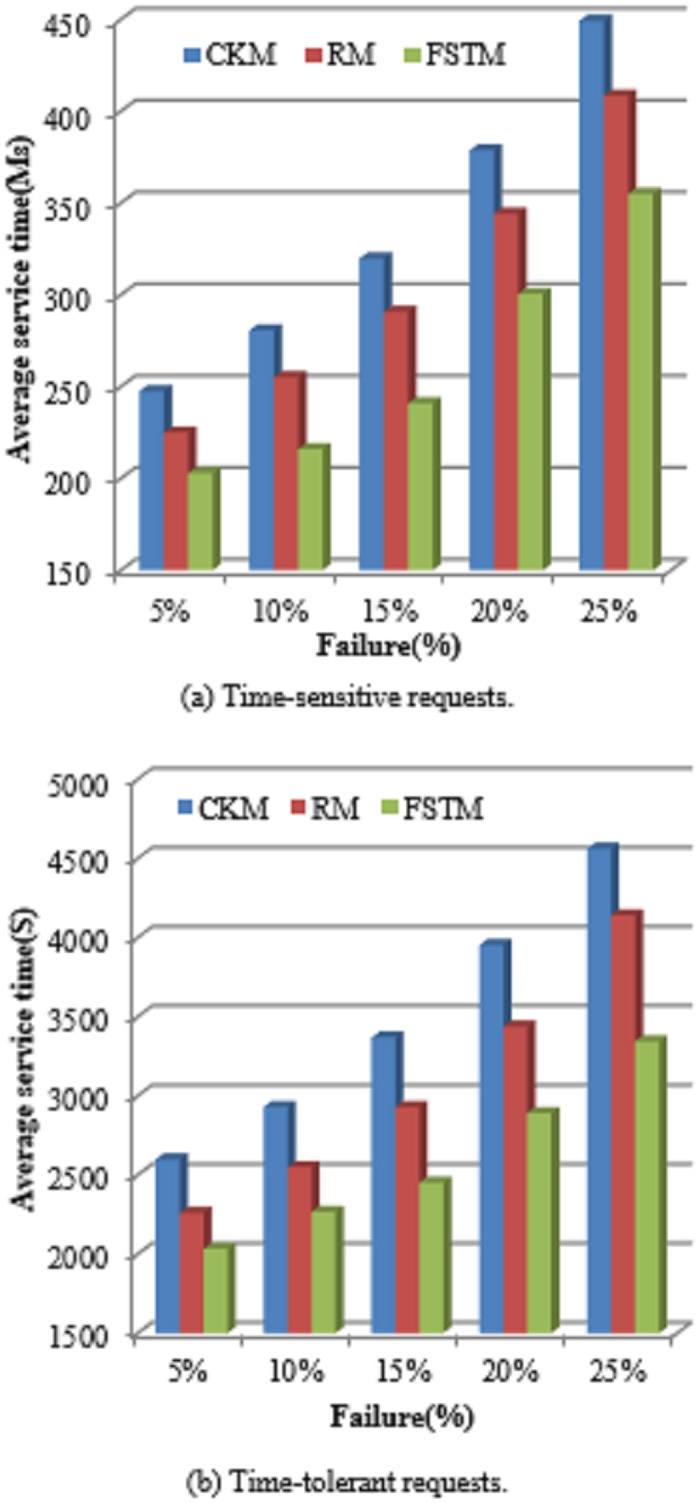
Average service time comparison.

The service time is the first important indicator for most customers. The service time of a request represents the amount of time spent from sending the request until completing it. Fig 7 shows the comparison of the average service time between the FTSM method and both the CKM and RM methods. Fig 7(a) shows the comparison for time-sensitive requests and Fig 7(b) shows the comparison for time-tolerant requests. It is obvious that as the number of requests increases the average service time increases for the three methods. However, it is clearly shown that the FTSM method provides smaller average service time compared to the other two methods. This is because the FTSM method prepares a predefined list of devices for each time-sensitive request and each incoming request is directly mapped to the suitable actuator. This means that the time overheads expended in the request scheduling, denoted as *τ*_*sh*_ in Eq (10), will be avoided because scheduling was done before the advent of the request. Therefore, the service time of the FTSM method is reduced. For time-tolerant requests, both RM and CKM use a single technique of fault tolerance. This causes the unavailability of suitable resources for serving some requests and then these requests should wait for these suitable resources to become free. Thus, the service time of such requests is prolonged.

#### 5.2.2 Throughput

Throughput is an important indicator of the productivity rate of a computing system. In the fog-cloud computing environment, it is represented as the number of successfully completed requests in a specified period of time.

$$Throughput=\frac{no.of\;requests\;successfully\;completed\;}{the\;specified\;period\;of\;time}.$$ (14)

**Fig 8:**
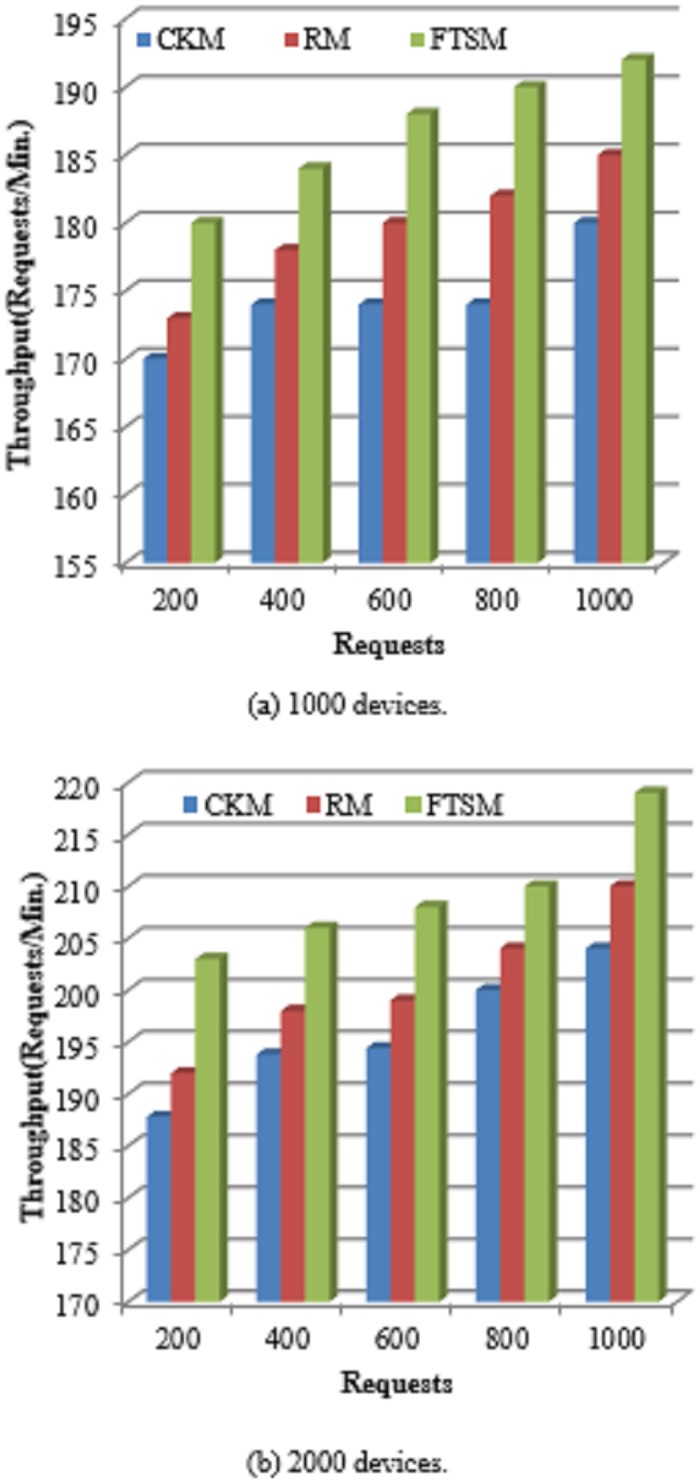
Throughput comparison.

Customers need to get services for their requests in the minimal possible time. So, as the throughput increases the customer satisfaction increases. Fig 8 indicates the throughput resulting from applying the FTSM, CKM and the RM methods. As the number of requests increases the throughput increases for the three methods. However, it is clearly shown that the FTSM method provides better throughput compared to the other two methods. This is also because the FTSM method prepares a predefined list of devices for each time-sensitive request and each incoming request is directly mapped to the suitable actuator. This means that the time overheads expended in the request scheduling, denoted as *τ*_*sh*_ in Eq (10), will be avoided because scheduling was done before the advent of the request. Therefore, the overall response time of the FTSM method is reduced and then the throughput is increased.

In RM method, there will be some times that there are no available backup resources for the request and then the request will wait for a suitable backup device to be available. This will prolong the service time for the request. In CKM, if a failure occurred the request should restart from the last checkpoint saved. Also, this will prolong the service time for the request.

#### 5.2.3 Operation costs

**Fig 9:**
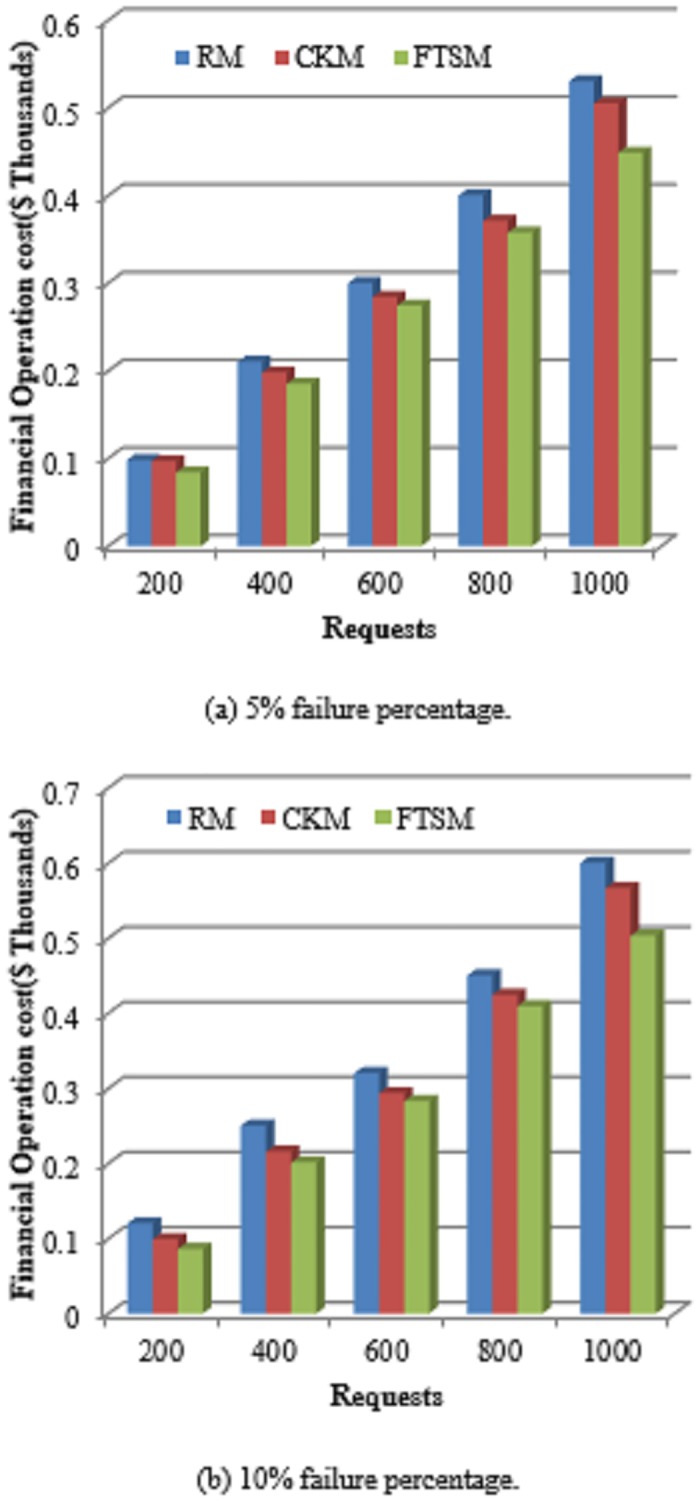
Operation costs comparison.

Operation costs represent a substantial issue for service providers. From the providers’ perspective, it is important to reduce the operation costs of serving requests to the most possible extent in order to keep their profits unaffected. This will allow providers to extend their working environments and to continuously update the underlying platforms. In addition, the providers can serve more requests and then their revenues increase. Fig 9 shows the comparison of the Operation costs between the FTSM method and both the CKM and RM methods. As the number of requests increases the cost increases for the three methods. However, it is clearly shown that the FTSM method provides better Operation costs compared to the other two methods. This is because the FTSM method depends on using multiple techniques for applying fault tolerance instead of applying only the checkpoint technique as in the CKM method or the replication technique as in the RM method.

The FTSM method is better than the RM method because the FTSM selects a suitable fault-tolerant technique from replication, checkpointing and resubmission according to the time sensitivity of requests and the conditions of the environment. This will minimize the number of devices used in tolerating failures and then these devices can be used to serve other requests and thus Operation costs are saved. However, the RM method applies only the replication method for all types of requests. This would exhaust the cloud resources in replication and would cause a revenue loss that would be gained from using these resources.

On the other hand, the CKM method applies only the checkpointing technique for all types of requests. The checkpointing technique would be unsuitable for some types of requests, especially time-sensitive requests. This is because each request is served by only one device and in case of failure, the request will be resumed from the last checkpoint on the same or another device. This will need more resources to be exhausted in the preparation of resumption, such as memory and bandwidth. Therefore, the operation costs increase.

In addition, the FTSM uses a variant checkpoint interval for each device which is determined based on the standard deviation of the operation time between failures (OTBF) rather than the constant checkpoint interval that CKM uses. Also, the status of the device is stored on the device that will complete the execution of the request in case the primary device fails for a long time. So, migration and time overheads could be minimized which can indirectly save money.

#### 5.2.4 Success rate

The success rate is an important performance indicator for evaluating the reliability of computing systems. As the success rate increases the reliability increases. It refers to the ratio of completed requests within the required due time to the total number of requests within a certain period of time.

$$Success\;rate=\frac{no.of\;requests\;successfully\;completed\;}{Total\;no.\;of\;requests}.$$ (15)

**Fig 10:**
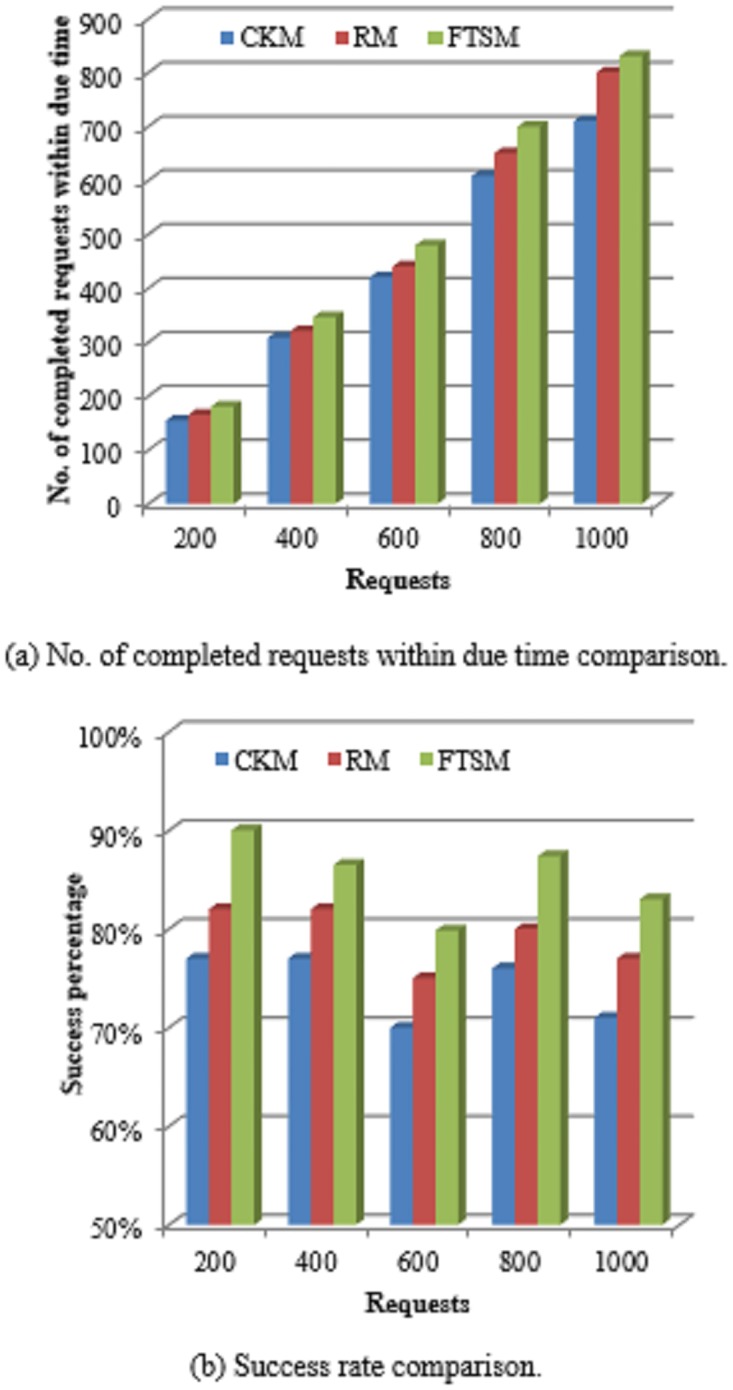
Completed requests comparison.

Fig 10 shows the comparison between the FTSM method and both the CKM and the RM methods in terms of the number of completed requests within the due time. As the number of requests increases the number of completed requests within the due time increases for the three methods. However, it is obvious that the FTSM method has a higher number of completed requests within due time than the other two methods. This is because the FTSM method provides smaller response times than the other two methods due to the exclusion of the scheduling time for the time-sensitive requests. Also, the FTSM method selects a suitable fault-tolerant technique for each request. This allows a great chance for requests to be completed within the required due times.

#### 5.2.5 Capacity percentage

The capacity of computing systems indicates their ability to take in and serve more requests with high reliability. Failure probability increases as the cloud get closer to its full capacity. This is because tolerating faults needs additional resources to be used and as the cloud get closer to its full capacity the number of available resources decreases. Thus, resources may not be available for fulfilling fault tolerance. So, it is crucial to preserve the capacity of the cloud by employing efficient methods that use a less number of resources without affecting the performance in terms of quality of service required. In addition, this will allow more requests to be served by the saved resources.

**Fig 11:**
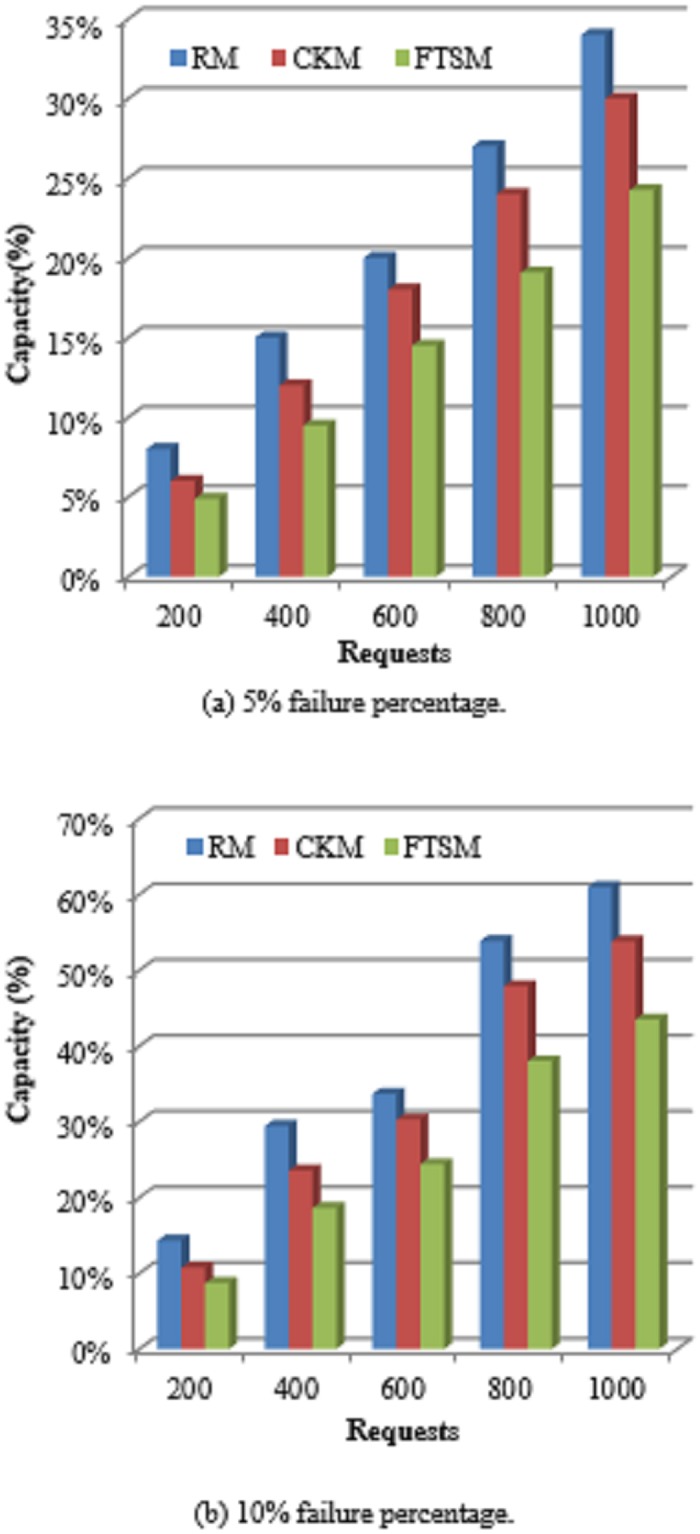
Capacity percentage comparison.

Fig 11 shows the comparison between the FTSM method and both the CKM and RM methods in terms of the percentage of cloud capacity used. As the number of requests increases the percentage usage of the capacity increases for the three methods. However, it is clearly shown that the FTSM method has a lower capacity usage than the other two methods. This is by cause of applying more than one fault tolerance techniques such as replication, checkpointing and resubmission by the FTSM method. In contrast, the RM method applies only replication technique for all types of requests. This will add a substantial amount of resources because not all requests have high importance so that they need replication. From the same point, the CKM method applies only the checkpointing technique for all types of requests. Some cloud resources will be exhausted in serving checkpoints of fiddling requests while resubmission technique could be sufficient for this type of requests.

#### 5.2.6 Availability

The service availability is referred to as the percentage of uptime to the total time of the cloud. The uptime is the time the cloud works properly without affected by the failures occurrence. The total time is the sum of the uptime and downtime or failure duration. The availability can be expressed by:

$$Availability(\%)=\frac{uptime}{total\;time}.$$ (16)

**Fig 12:**
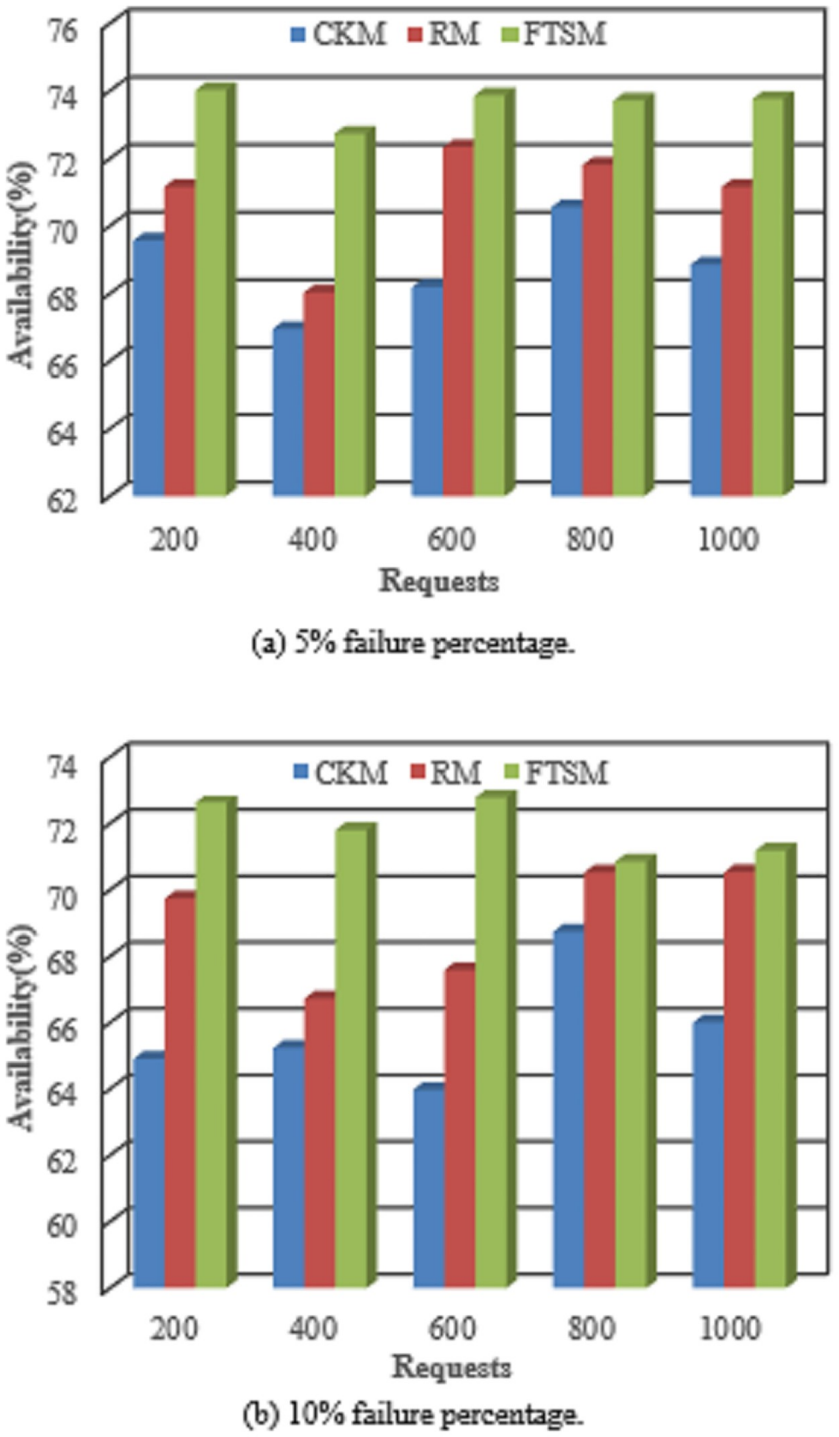
The availability comparison.

Fig 12 shows the comparison between the FTSM method and both the CKM and the RM methods in terms of the availability. However, it is obvious that the FTSM method has a better availability than the other two methods. This is because the FTSM method employs variant checkpoint interval and number of replications according to the category of the request. This will help to reduce the failure numbers.

## 6 Conclusions

In order to leverage the concept of fog or edge computing, failures should be highly tolerated. A fault-tolerant allocation method for allocating services’ requests of devices at the cloud edge to the most appropriate resources is presented and assessed in this paper. The method classifies devices that issue requests according to their needs of time and resources. For time-sensitive requests, a pre-prepared list of executive devices is accessed and each request is directly dispatched to suitable actuators.

The performance of the method is evaluated with a replication-based method and also with a checkpointing-based method in terms of average service time, throughput, Operation costs, success rate and capacity percentage. Experimental results indicate that the proposed framework improves the cloud’s performance as shown in Table 3.

**Table 3:** Improvement in performance.

Metric	The improvement over CKM(%)	The improvement over RM(%)
Average service time	30%	16%
Throughput	7%	5%
Operation costs	8%	10%
Success rate	11%	9%
Capacity percentage	4%	6%
Availability	8%	4%

The main findings of the proposed method are represented in the minimization of the latency and overheads of services and to the increase of the reliability and capacity of the cloud. Additionally, the method provides better levels of availability of the cloud resources. For the provider’s perspective, these findings will improve the reputation and increase the profit. For the customer’s perspective, the findings reveal that customers will enjoy a distinctive level of service quality in terms of response time and cost.

Next, we plan to extend our research to include other types of failures encountered during forward and backward transmission. In addition, we plan to focus on optimizing the amount of consumed electrical power.

## Supporting information

S1 File Request200. (TXT)

S2 File Request400. (TXT)

S3 File Request600. (TXT)

S4 File Request800. (TXT)

S5 File Request1000. (TXT)
